# D-dimer levels and cerebral infarction in critically ill cancer patients

**DOI:** 10.1186/s12885-017-3588-7

**Published:** 2017-08-30

**Authors:** Jeong-Am Ryu, Oh Young Bang, Geun-Ho Lee

**Affiliations:** 1 Department of Critical Care Medicine, Samsung Medical Center, Sungkyunkwan University School of Medicine, 81 Irwon-ro, Gangnam-gu, Seoul, 135-710 Republic of Korea; 2 Department of Neurology, Samsung Medical Center, Sungkyunkwan University School of Medicine, 81 Irwon-ro, Gangnam-gu, Seoul, 135-710 Republic of Korea; 3 Department of Neurology, Dankook University College of Medicine, Anseo-dong San 16-5, Cheonan-si, Chungcheongnam-do 330-715 Republic of Korea

**Keywords:** D-dimer, Cerebral infarction, Cancer, Brain magnetic resonance imaging, Intensive care unit

## Abstract

**Background:**

D-dimer levels have been used in the diagnosis of a variety of thrombosis-related diseases. In this study, we evaluated whether measuring D-dimer levels can help to diagnose cerebral infarction (CI) in critically ill cancer patients.

**Methods:**

We retrospectively evaluated all cancer patients who underwent brain magnetic resonance imaging (MRI) between March 2010 and February 2014 at the medical oncology intensive care unit (ICU) of Samsung Medical Center. Brain MRI scanning was performed when CI was suspected due to acute neurological deficits. We compared D-dimer levels between patients ultimately diagnosed as having or not having CI and analyzed diffusion-weighted imaging (DWI) lesion patterns.

**Results:**

A total of 88 patients underwent brain MRI scanning due to clinical suspicion of CI; altered mental status and unilateral hemiparesis were the most common neurological deficits. CI was ultimately diagnosed in 43 (49%) patients. According to the DWI patterns, multiple arterial infarctions (40%) were more common than single arterial infarctions (9%). Cryptogenic stroke etiologies were more common (63%) than determined etiologies. There was no significant difference in D-dimer levels between patients with and without CI (*P* = 0.319). Although D-dimer levels were not helpful in diagnosing CI, D-dimer levels were associated with cryptogenic etiologies in critically ill cancer patients; D-dimer levels were higher in the cryptogenic etiology group than in the determined etiology group or the non-infarction group (*P* = 0.001). In multivariate analysis, elevated D-dimer levels (> 8.89 μg/mL) were only associated with cryptogenic stroke (adjusted OR 5.46; 95% confidence interval, 1.876–15.857).

**Conclusions:**

Abnormal D-dimer levels may support the diagnosis of cryptogenic stroke in critically ill cancer patients.

## Background

Cancer patients are especially prone to stroke [1]. In an autopsy study of patients with malignancy, 7% had shown clinical symptoms while 15% had pathologic evidence of stroke [2]. Cancer is associated with cerebral infarction (CI) via various mechanisms, including coagulation disorders and tumor occlusion [3–8]. Furthermore, cancer-associated hypercoagulability may be important in CI without conventional stroke mechanisms [9].

Elevated plasma D-dimer levels may be observed in various conditions and in critically ill patients [10, 11]. D-dimer measurements have been used to diagnosis of a variety of thrombosis-related diseases [10], and previous studies have shown an association between elevated D-dimer levels and cancer-related CI [8, 9, 12]. Furthermore, elevated D-dimer levels and multiple territorial ischemic lesions may be predictive factors in cancer-related CI [8]. However, to the best of our knowledge, there have been no reports on the predictive value of CI in critically ill cancer patients. In addition, it remains unknown whether D-dimer levels are helpful in distinguishing between CI and non-infarction in critically ill cancer patients.

In this study, we evaluated whether measuring D-dimer levels can help to diagnose CI in critically ill cancer patients with acute neurological deficits during their stay in the intensive care unit (ICU).

## Methods

This retrospective observational study evaluated cancer patients in the medical oncology ICU of Samsung Medical Center (a 1961-bed, university-affiliated, tertiary referral hospital in Seoul, South Korea) between March 2010 and February 2014. This study was approved by the Institutional Review Board of Samsung Medical Center according to the Declaration of Helsinki on reviewing and publishing information from patient’s records. Informed consent was waived due to the retrospective nature of the study.

### Patients

Subjects were eligible for this study if they were at least 18 years of age, diagnosed with either solid tumors or hematologic malignancies, and were admitted to the medical oncology ICU of Samsung Medical Center. All included patients underwent brain magnetic resonance imaging (MRI) during their ICU stay. Brain MRI was performed when CI was suspected due to acute neurological deficits. Patients were excluded if they had a history of head trauma, neurosurgery, or a chronic neurological abnormality.

### Data collection

We reviewed clinical and laboratory data, including conventional stroke risk factors, malignancy type and status, and neurological abnormalities at the time of brain MRI scanning. D-dimer levels were collected within 48 h of the brain MRI scans; when multiple samples were available, we used samples obtained closest to the time of the scan. The immunoturbidimetric assay is a second-generation automated latex agglutination assay that uses specialized analyzers to record the rate at which antibody-coated particles aggregate in response to D-dimer antigen. The normal range of D-dimer levels at our institution was 0 to 0.50 μg/mL, the analytical measurement range was 0.27 to 4.00 μg/mL, and the clinically reportable range was 0.01 to 60.00 μg/mL [13]. Only cancer patients were included in this study, and all definitions associated with cancer status have been previously reported. Cancer status was classified as either first presentation, relapsed/refractory, extensive disease, or major organ involvement [14–17].

Brain MRI scans were performed using a 1.5 T (Signa Advantage Horizon, GE Medical Systems, Milwaukee, WI, USA) with quadrature head coils from 1997 to 2010 and 3 T (Achieva, Philips Healthcare, Best, the Netherlands) from 2006 to 2014 with eight-channel phased-array head coils. The brain MRI scans were independently read by two neurologists and one neuroradiologist; investigators used commercial image-viewing software (Centricity RA1000 PACS Viewer; GE Healthcare, Milwaukee, Wisconsin, USA). CI was diagnosed by ischemic regions with decreased apparent diffusion coefficients (ADCs) and high signal intensities on diffusion-weighted imaging (DWI) [18]. DWI patterns were defined as single arterial infarction or multiple arterial infarctions. The stroke subtype was classified according to the Trial of Org 10,172 in the Acute Stroke Treatment (TOAST) system [19]. Stroke etiology was classified as (1) large artery atherosclerosis when there was large vessel disease (stenosis >50%) responsible for ischemic lesions without cardioembolic sources or lacunar infarction; (2) cardioembolism when a cardioembolic source was present without evidence of large or small vessel disease; (3) small vessel occlusion when there were subcortical infarcts (< 15 mm in diameter) without an embolic source in the heart or parent large vessels; (4) other rare etiologies (arterial dissection, moyamoya disease, etc.); and (5) cryptogenic (undetermined) when no etiologies could be identified [7, 19]. To determine the causes of stroke, patients underwent cardiac evaluation (transthoracic echocardiography [TTE], transesophageal echocardiography [TEE]), studies of the intracranial arteries and neck vessels (non-contrast enhanced-MRA [NCE MRA], contrast-enhanced MRA [CE MRA], CT angiography [CTA]), and transcranial Doppler (TCD) for detection of patent foramen ovale or microembolic signal. All strokes and stroke etiologies in this study were diagnosed in consultation with a neurologist.

### Statistical analyses

All data are presented as medians and interquartile ranges (IQRs) for continuous variables or as numbers (percentages) for categorical variables. The predictive performances of D-dimer levels and DWI patterns were assessed using the area under the curve (AUC) of receiver operating characteristic (ROC) curves of [sensitivity / (1-specificity)]. AUCs were compared using the nonparametric approach of DeLong et al. [20] for two correlated AUCs. The optimal cut-off values of D-dimer levels for predicting cryptogenic stroke were obtained by ROC curve and Youden index [21, 22]. Data were compared using the Kruskal-Wallis test and the Mann-Whitney U test for continuous variables, and the chi-square test or Fisher exact test for categorical variables. Multiple logistic regression analysis was used to identify independent predictors of CI in critically ill cancer patients; the estimated odds ratio (OR) and 95% confidence interval (CI) for each parameter were calculated. Variables with a *P* value < .05 on the univariate analysis, as well as a priori variables that were clinically relevant, were entered into the forward stepwise multiple logistic regression model. All tests were two-sided, and *P* values < .05 were considered significant. All data were analyzed using the Statistical Package for the Social Science software version 20.0 (IBM, Armonk, NY, USA).

## Results

A total of 2258 critically ill cancer patients were admitted to the medical oncology ICU from March 2010 to February 2014. Of these, 88 cancer patients were enrolled in the final analysis. All of these patients underwent brain MRI because they were suspected of having CI due to acute neurological deficits during their ICU stay.

Baseline characteristics of these 88 patients are presented in Table 1. The median age was 63 (IQR 53–69) years, and 51 patients (58%) were male. Of these 88 patients, 33 (38%) had solid tumors, including lung cancer (*n* = 18), hepatic cancer (*n* = 3), gastric cancer (*n* = 3), brain cancer (*n* = 2), and other solid tumors (*n* = 7). The remaining 55 (63%) patients had hematologic malignancies, including leukemia (*n* = 18), lymphoma (*n* = 22), multiple myeloma (*n* = 10), myelodysplastic syndrome (*n* = 3), and other hematologic malignancies (*n* = 2). Thirty-three (38%) patients were classified as first presentation, 45 (51%) as relapsed/refractory, 38 (43%) as extensive disease, and 21 (24%) as major organ involvement including the brain in 10, the lung in 7, and the liver in 4. The most common reason for ICU admission was respiratory failure (42%). The most common vascular risk factors for CI were hypertension (40%) and smoking (35%). Altered mental status was the most common neurological deficit for clinically suspicious CI, which was present in 55 (63%) patients; this was followed by unilateral hemiparesis in 28 (32%), seizure in 20 (23%), abnormal involuntary movement in 6 (7%), and anisocoric pupil or abnormal pupil reflex in 3 (3%) patients. Overlap of these neurological deficits was present in 33% of patients. The median interval from brain MRI scan to D-dimer measurement was −5.0 (IQR -14.9-0.1) hours. The median interval from symptom onset to D-dimer measurement was 5.6 (IQR 0.2–36.0) hours. Although TTE was performed in about two-thirds of ischemic strokes (29 patients), TEE was performed in only 10 patients (6 cryptogenic and 4 cardioembolic). Studies of the neck and intracranial vessels were performed in 34 stroke patients (79%, NCE MRA 2, CE MRA 31, CTA 1). TCDs were performed in 7 stroke patients (16%).

**Table 1:** Clinical characteristics of 88 critically ill cancer patients at the time of brain MRI for clinical suspicion of cerebral infarction in the ICU

Characteristics	No. of patients (%) or median (IQR)
Age, years	63 (53–69)
Gender, male	51 (58)
Type of malignancy	
Solid tumor	33 (38)
Hematologic	55 (63)
Vascular risk factors	
Hypertension	35 (40)
Diabetes mellitus	20 (23)
Past and current smoking	31 (35)
Ischemic heart disease	3 (3)
Hypercholesterolemia	5 (6)
Atrial fibrillation	19 (22)
Alcohol abuse	4 (5)
Family history of stroke	7 (8)
Previous thrombotic event	10 (11)
Ischemic stroke	3 (3)
Myocardial infarction	4 (5)
Deep vein thrombosis	2 (2)
Pulmonary thromboembolism	1 (1)
Neurological deficits	
Decreased mentality or delirium	55 (63)
Hemiparesis	28 (32)
Seizure	20 (23)
Abnormal movement	6 (7)
Anisocoric pupil or abnormal pupil reflex	3 (3)
Abnormal respiratory pattern	2 (2)
Other	7 (8)
Time interval from ICU admission to brain MRI, days	4.4 (1.1–12.4)
Recent chemotherapy	44 (50)
Anticoagulation use	20 (23)
Antiplatelet use	3 (3)
DIC	3 (3)
D-dimer levels (μg/mL)	4.55 (2.66–11.15)
Fibrinogen	327 (195–477)
CRP	6.43 (3.29–15.95)
Procalcitonin	1.05 (0.27–3.77)

*IQR* interquartile range, *ICU* intensive care unit, *MRI* magnetic resonance imaging, *DIC* disseminated intravascular coagulation, *CRP* C-reactive protein

The median interval between the initial cancer diagnosis and the brain MRI scan was 125 (IQR 28–437) days. There was no significant difference in the median interval between patients with ischemic and non-ischemic stroke (144 [27–536] days vs. 102 [28–312] days, *P* = 0.815). The brain MRI findings of these 88 patients are summarized in Table 2. Forty-three (49%) patients were ultimately diagnosed with CI, 7 (8%) with new central nerve system metastases, and 13 (15%) had normal brain MRI scans. Multiple arterial infarctions (40%) were more common than single arterial infarctions (9%). Stroke etiologies were identified in 16 (37%) patients, including cardioembolism (*n* = 8), large vessel atherosclerosis (*n* = 3), small vessel occlusion (*n* = 2), and other (*n* = 3). However, the remaining 27 (63%) patients had no determined etiologies (cryptogenic stroke).

**Table 2:** Brain MRI findings in 43 patients diagnosed with cerebral infarction and 45 with non-infarction during their stay in the ICU

Brain MRI findings	No. of patients (%)
Cerebral infarction	
Single arterial lesion in DWI	8 (9)
Multiple arterial lesions in DWI	35 (40)
Small lesions involving multiple arterial territories	18 (20)
Small and large disseminated lesions	17 (19)
Non-infarction	
Pathologic brain MRI findings	32 (36)
CNS metastasis^a^	7 (8)
Old stroke lesion	7 (8)
Posterior reversible encephalopathy syndrome	4 (5)
Intracranial hemorrhage (1 gyral SAH, 2 SDH)	3 (3)
Seizure-related change	3 (3)
Primary brain tumour	3 (3)
Other	5 (6)
Normal brain MRI findings	13 (15)

*MRI* magnetic resonance imaging, *DWI* diffusion-weighted imaging, *CNS* central nerve system, *SAH* subarachnoid hemorrhage, *SDH* subdural hemorrhage

^a^Newly diagnosed CNS metastasis

There was no significant difference in D-dimer levels between the CI group and the non-infarction group (*P* = 0.319). After adjusting for potential confounding factors, D-dimer measurements were not helpful in confirming the presence of CI among critically ill cancer presenting with neurological deficits (adjusted OR 1.08; 95% CI, 0.998–1.170).

**Fig. 1:**
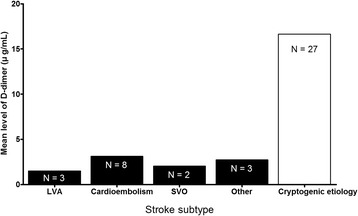
Stroke subtype and D-dimer levels. LVA, large artery atherosclerosis; SVO, small vessel occlusion

Although D-dimer levels were not helpful in diagnosing CI, they were associated with cryptogenic etiologies in critically ill cancer patients. D-dimer levels were higher in stroke patients with cryptogenic etiology than determined etiologies. Stroke subtypes and D-dimer levels are depicted in Fig. 1. We then re-analyzed and classified the study subjects into non-infarction, determined etiology, or cryptogenic etiology groups. Univariate comparisons of each group are presented in Table 3. There were no significant differences with respect to sex, malignancy type, recent chemotherapy, vascular risk factors, use of antiplatelet and anticoagulant drugs, or fibrinogen or procalcitonin levels between the three groups at the time that CI was suspected. With respect to DWI patterns, multiple arterial infarctions were more common in the cryptogenic etiology group than in the determined etiology group (*P* = 0.002). D-dimer levels were higher in the cryptogenic etiology group than in the determined etiology group or the non-infarction group (*P* = 0.001). D-dimer levels >8.89 μg/mL predicted cryptogenic stoke with a sensitivity of 60% (95% CI, 38.8–77.6%) and a specificity of 83% (95% CI, 69.7–91.8%) in patients with acute neurological deficits. In multivariate analysis, elevated D-dimer levels (> 8.89 μg/mL) were only associated with cryptogenic etiologies among patients who underwent brain MRI for suspected CI in the medical oncology ICU (adjusted OR 5.46; 95% CI, 1.876–15.875). In addition, D-dimer levels >6.28 μg/mL in patients with hematologic malignancies predicted cryptogenic stroke with a sensitivity of 67% (95% CI, 38.4–88.2%) and a specificity of 76% (95% CI, 58.8–89.3%). D-dimer levels >8.53 μg/mL in patients with solid tumors predicted cryptogenic stroke with a sensitivity of 73% (95% CI, 39.0–94.0%) and a specificity of 78% (95% CI, 52.4–93.6%).

**Table 3:** Comparisons of clinical characteristics at the time of brain MRI for clinical suspicion of cerebral infarction and outcomes between non-infarction group, determined stroke etiology group, and cryptogenic stroke etiology group

	Non-infarction group (*n* = 45)	Determined etiology group (*n* = 16)	Cryptogenic etiology group (*n* = 27)	*P* value
Age, years	63.0 (41.0–69.0)^a^	67.5 (58.0–69.5)^a^	64 (58.5–69.0)^a^	0.278
Gender, male	25 (56)	12 (75)	14 (52)	0.297
Type of malignancy				
Solid tumor	17 (38)	4 (25)	12 (44)	0.444
Hematologic	28 (62)	12 (75)	15 (56)	
Recent chemotherapy	22 (49)	9 (56)	13 (48)	0.857
Vascular risk factors				
Hypertension	18 (40)	5 (31)	12 (44)	0.693
Past and current smoking	17 (38)	6 (38)	8 (30)	0.765
Ischemic heart disease	1 (2)	1 (6)	1 (4)	0.744
Hypercholesterolemia	3 (7)	1 (4)	3 (7)	0.866
Atrial fibrillation	11 (24)	5 (31)	3 (11)	0.241
Diabetes mellitus	9 (20)	3 (19)	8 (30)	0.586
Alcohol abuse	1 (2)	2 (13)	1 (4)	0.230
Thrombotic event	3 (7)	5 (31)	5 (19)	0.047
Previous thrombotic event^b^	n (7)	3 (19)	4 (15)	0.338
Concomitant pulmonary thromboembolism	0 (0)	0 (0)	2 (7)	0.099
Concomitant deep vein thrombosis	1 (2)	4 (25)	1 (4)	0.006
DWI pattern				0.002
Single arterial infarction		7 (44)	1 (4)	
Multiple arterial infarction		9 (56)	26 (96)	
Antiplatelet use	1 (2)	1 (6)	1 (4)	0.744
Anticoagulant use	10 (22)	3 (19)	7 (26)	0.857
DIC	0 (0)	1 (6)	2 (7)	0.193
D-dimer (μg/mL)	4.86 (2.30–8.53)^c^	2.66 (2.29–3.75)^c^	10.38 (3.92–21.21)	0.001
Fibrinogen	323 (167–441)^d^	322 (262–433)^d^	358 (267–507)^d^	0.707
CRP	4.79 (3.24–7.13)^e^	11.66 (1.52–14.90)^e,f^	15.48 (6.42–22.44)^f^	0.036
Procalcitonin	1.05 (0.31–2.17)^g^	2.05 (0.45–3.52)^g^	0.80 (0.22–4.22)^g^	0.560
Outcomes				
ICU mortality	13 (29)	7 (44)	12 (46)	0.283
In-hospital mortality	29 (64)	13 (81)	21 (81)	0.227
Length of stay in ICU, days	13.6 (6.7–21.8)^h^	18.3 (8.7–36.5)^h^	14.7 (9.2–19.2)^h^	0.381

Data are expressed as medians (interquartile range) or frequencies (%)

Statistical significances of continuous variables were tested by the Kruskal-Wallis test among groups

The same letters indicate non-significant differences between groups base on the Mann-Whitney U test

*DWI* diffusion-weighted imaging, *DIC* disseminated intravascular coagulation, *CRP* C-reactive protein, *ICU* intensive care unit

^b^Previous thrombotic events include ischemic stroke, myocardial infarction, deep vein thrombosis, and pulmonary embolism

**Fig. 2:**
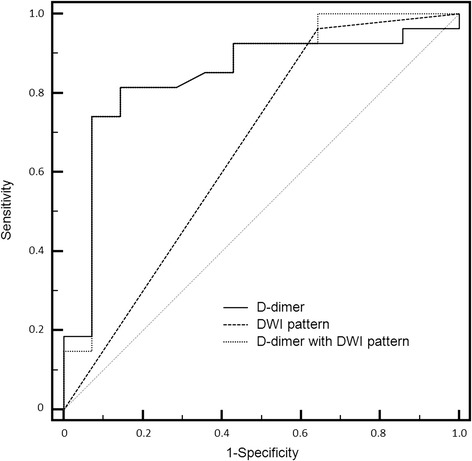
Receiver operating characteristic (ROC) curves for D-dimer levels, diffusion-weighted imaging (DWI) patterns, and D-dimer levels with DWI patterns to predict cryptogenic stroke etiologies

To determine which marker better predicted CI, we compared models with D-dimer levels and DWI patterns separately before combining them into a single model. When evaluated separately, the predictive power of D-dimer levels was better than DWI patterns. The ROC curves for D-dimer levels and DWI patterns predicted cryptogenic stroke with AUCs of 0.837 (0.689–0.934) and 0.660 (0.496–0.800), respectively. However, the predictive power of D-dimer levels combined with DWI patterns (AUC of 0.856 [0.711–0.946]) was similar to that of D-dimer levels alone (Fig. 2). Elevated D-dimer levels (> 3.92 μg/mL) also help to distinguish cryptogenic etiologies from determined etiologies (sensitivity 74% [95% CI, 53.7–88.9%], specificity 93% [95% CI, 66.1–99.8%]).

There were no significant differences in the length of ICU stay (*P* = 0.381), ICU mortality (*P* = 0.283), or in-hospital mortality (*P* = 0.227) between the three groups.

## Discussion

Here we evaluated whether D-dimer measurements were helpful in diagnosing CI in cancer patients suspected of having CI due to acute neurological deficits during their ICU stay. Although measuring D-dimer levels was not helpful in the diagnosis of CI, it might be helpful in distinguishing between determined etiology and cryptogenic etiology in critically ill cancer patients with CI. Approximately half of the patients in this study who underwent brain MRI for acute neurological deficits were ultimately diagnosed with CI, and cryptogenic etiologies were more common than determined etiologies. Multiple arterial infarctions were more commonly observed in the DWI patterns. While the majority of cryptogenic strokes showed this multiple arterial infarction pattern, it was difficult to distinguish stroke etiologies by DWI patterns alone.

There are many clinical conditions characterized by elevated D-dimer levels, including disseminated intravascular coagulation, venous thromboembolism, ischemic cardiomyopathy, stroke, trauma, burn, sepsis, and cancer, among others [10]. D-dimer is nonspecific and may be elevated in both cancer patients and critically ill patients [10]; however, D-dimer measurements may be helpful for the diagnosis and management of various thrombosis-related diseases [10]. Malignancies are associated with hypercoagulable and prothrombotic states due to the ability of tumor cells to activate the coagulation system [23]. In addition, D-dimer levels are significantly associated with the activity and prognosis of malignancies [24–26]. Previous studies have reported an association between elevated D-dimer levels and cancer-related stroke [6, 8, 12]. Elevated D-dimer levels have been more commonly observed in CI with malignancies than in CI without malignancies [6, 8, 12, 27]. Furthermore, D-dimer measurements might be useful for screening malignancies in stroke patients [12]. In this study, elevated D-dimer levels were more closely associated with strokes with cryptogenic etiology than with determined etiology. Although measuring D-dimer levels was not helpful in diagnosing CI itself, it might be useful in distinguishing between strokes with cryptogenic and determined etiology when critically ill cancer patients present with acute neurological deficits.

Several studies have reported that multiple vascular territorial CI may be associated with cancer-related stroke [6, 8, 12]. Furthermore, elevated D-dimer levels and multiple arterial infarctions have been reported to be independent predictors of cancer-related stroke [8, 27]. In this study, most cryptogenic strokes showed multiple territorial lesions, and the DWI patterns were different between stroke etiology groups; however, it was difficult to distinguish stroke etiologies based on brain DWI patterns alone. Over 50% of patients with determined stroke etiology also had multiple territorial lesions. In addition, cardioembolism was most common in patients with determined etiologies, and multiple arterial lesions were commonly observed in patients with cardioembolic stroke.

Cancer-associated hypercoagulability can lead to CI. A hypercoagulable state can result from metastatic lesions to the brain or from vascular injury due to cancer therapy [5–7, 28]. Recent studies reported strokes with malignancy differ from strokes without malignancy in terms of risk factors, stroke patterns, and stroke mechanisms [7, 8]. Most stroke etiologies were not consistent with known conventional stroke mechanisms in this study, which matches previous reports. In cancer patients without determined etiologies, cancer-specific mechanisms can be considered to be the main cause of stroke [6]. Consequently, most cryptogenic strokes in this study are presumably cancer-related.

This study had several limitations, in part due to its retrospective design. Also, this study was conducted at a single institution with a specialized medical ICU for critically ill cancer patients. Therefore, the results of our study may not be widely applicable to other centers in which there are no experienced intensivists available for oncological critical care. Another limitation is the fact that we did not systematically screen patients with acute neurological deficits for the prevalence of stroke during their ICU stay. Therefore, selection bias might have been an influence. In addition, there was no routine screening for deep vein thrombosis or pulmonary thromboembolism in this study. Cancer-related hypercoagulability might be associated with concomitant thromboembolism, and non-symptomatic thromboembolism might have been under evaluated. Although the cardiac status of most patients was evaluated by TTE, TEE was only performed in a limited number of cases because it is somewhat invasive. TTE, however, was insufficient to find intracardiac thrombus or nonbacterial thrombotic endocarditis (NBTE). No cases of NBTE were detected in this study; however, this could be due to the limited use of TEE. Finally, more severely ill patients might not have undergone brain MRI, even if they were suspected of having CI. The number of patients with acute neurological deficits who refused further evaluation could not be determined from the medical records of the study period.

## Conclusions

D-dimer measurements were not helpful in diagnosing CI in critically ill cancer patients suspected of having CI due to acute neurological deficits. Abnormal D-dimer levels may be observed in various conditions and in critically ill patients, and may be specifically associated with cancer-related stroke. It is therefore reasonable to suspect cancer-related stroke when elevated D-dimer levels are accompanied by acute neurological deficits in critically ill cancer patients.

## Abbreviations

ADC: Apparent diffusion coefficients
AUC: Area under the curve
CE MRI: Contrast-enhanced magnetic resonance angiography
CI: Cerebral infarction
CTA: Computed tomography angiography
ICU: Intensive care unit
IQR: Interquartile range
MRI: Magnetic resonance imaging
NBTE: Nonbacterial thrombotic endocarditis
NCE MRA: Noncontrast-enhanced magnetic resonance angiography
OR: Odds ratio
ROC: Receiver operating characteristic
TCD: Transcranial Doppler
TEE: Transesophageal echocardiography
TOAST: Trial of Org 10,172 in Acute Stroke Treatment
TTE: Transthoracic echocardiography
